# Characteristic Correlation between Staphylococcal Isolates from Wounds and Nares of Diabetic Foot Patients Living in Erbil City, Iraq

**DOI:** 10.2174/0115733998413217251206210451

**Published:** 2026-03-26

**Authors:** Samira Fattah Hamid, Fatih Koksal, Tulin Guven Gokmen, Tulay Knademir

**Affiliations:** 1 Department of Medical Microbiology, College of Health Sciences, Hawler Medical University, Erbil, Iraq;; 2 Department of Clinical Microbiology, Faculty of Medicine, Cukurova University, Adana, Turkey;; 3 Department of Microbiology, Ceyhan Veterinary Faculty, Cukurova University, Adana, Turkey

**Keywords:** *Staphylococcus aureus*, coagulase-negative, *mecA* gene, genetic correlation, PFGE, Foot ulcers

## Abstract

**Introduction:**

Foot ulcers are a common and serious complication in patients with diabetes mellitus. Nasal carriage of methicillin-resistant *Staphylococcus aureus* (MRSA) may serve as a reservoir for autoinfection. This study aimed to investigate the association between *S. aureus* isolates obtained from nasal passages and foot infections in diabetic patients.

**Methods:**

Nasal and foot wound swabs were collected from 84 diabetic patients. Bacterial identification and antibiotic susceptibility testing were performed using the VITEK 2 system. Methicillin resistance was determined using Oxacillin Salt Agar Screen, PBP2a assays, and mecA gene detection. Genetic correlation between nasal and wound isolates was assessed by pulsed-field gel electrophoresis (PFGE).

**Results:**

*S. aureus* was the most prevalent isolate, accounting for 33.1% of samples. Methicillin resistance was detected in 55.1% of foot wound isolates and 73.3% of nasal isolates. The mecA gene was identified in 95.3% of both *S. aureus* and coagulase-negative *Staphylococci*. PFGE analysis showed 100% genetic homology between paired nasal and wound isolates.

**Discussion:**

The data confirmed that *S. aureus* is the predominant bacterium in diabetic foot lesions. Our findings also suggest that these infections in diabetic patients are likely of endogenous origin.

**Conclusion:**

Diabetic foot patients carrying *S. aureus* in the nares are at high risk of self-contamination. These findings highlight the need for routine nasal screening and decolonization strategies to reduce infection risk in this vulnerable population.

## 1 INTRODUCTION

Foot ulcer is an increasing problem among patients with diabetes mellitus and is considered the most common cause of hospitalization in diabetic patients [1]. *Staphylococcus* is the most common and virulent pathogen in diabetic ulcers. This pathogen is also a regular inhabitant of the skin microflora, colonizing the nares and other mucosa [2].

These sites serve as ecological niches from which bacteria can spread to other parts of the body, with significantly increased carriage rates observed in diabetic patients [3]. *Staphylococcus aureus* is the predominant pathogen in diabetic foot infections (DFIs), and a large proportion of isolates are methicillin-resistant. Patients infected with MRSA experience higher morbidity and mortality rates compared to those infected with susceptible strains [3-5].

Nasal carriage of methicillin-resistant *Staphylococcus aureus* (MRSA) is notable, as diabetes itself is a significant risk factor for colonization [6]. This nasal colonization serves as a potential reservoir, and eradication of *S*. *aureus* in the nares has been shown to reduce subsequent infections [7, 8].

The growth of MRSA in diabetic foot ulcers (DFUs) can impede wound healing, leading to frequent hospitalizations, repeated antimicrobial use, increased treatment failure, and a higher likelihood of surgical interventions, including limb amputation [9, 10]. The link between nasal colonization with *S. aureus* and the risk of endogenous infection is well documented [11, 12]. Previous studies have reported the recovery of identical *S. aureus* isolates from both the nares and ulcers of diabetic foot infection (DFI) patients, suggesting that nasal *S. aureus* colonization is a significant risk factor for DFI [13, 14].

To our knowledge, no study in Iraq has investigated the genetic similarity between wound and nasal isolates in diabetic foot patients. Assessment of anterior nares colonization allows for the early identification of DFU patients at risk of endogenous *S. aureus* infection. The implementation of targeted screening and decolonization strategies may be pivotal in preventing the progression of DFU to DFI. This study aimed to determine the prevalence of nasal carriage of *S. aureus* in diabetic foot patients and to assess its genetic correlation with *S. aureus* isolates recovered from DFI in the same patients.

## 2 MATERIALS AND METHODS

### 2.1 Sample Collection

In the current study, 84 patients with diabetic foot ulcers who were hospitalized between December 2016 and January 2018 at Rizgari and Hawler Teaching Hospitals in Erbil City, Iraq, were included (Table **S1**). The study was approved by the Ethics Committee of the College of Medicine at Hawler Medical University (Approval Code: 15, 23.04.2016), and all procedures were conducted in accordance with the Helsinki Declaration for research involving human subjects. Informed consent was obtained from all patients prior to specimen collection.

### 2.2 Inclusion and Exclusion Criteria

Patients with a confirmed diagnosis of diabetes mellitus, those who were incidentally diagnosed with diabetes after admission with a foot ulcer, and patients who provided informed consent were included in this study. Patients without diabetes, those with foot ulcers resulting from other causes, pregnant women, individuals younger than 18 years of age, and patients who declined to participate were excluded from the study.

### 2.3 Collection and Culture of Specimens

Specimens were collected aseptically from the nose and infected foot lesions of diabetic patients using sterile cotton swabs [15]. The samples were then cultured on blood agar and mannitol salt agar plates (Lab M / UK), incubated at 37°C, and examined after 24 and 48 hours.

### 2.4 Identification of Isolated Bacteria

After purification of the isolated bacteria, they were identified using the VITEK 2 system (BioMérieux Vitek, France) with a GP card.

### 2.5 Antibiotic Susceptibility Testing

According to the manufacturer’s instructions, the AST-GP580 Test Kit (REF 22233; BioMérieux Vitek, France) for Gram-positive bacteria was used to evaluate susceptibility to various antibiotics.

### 2.6 Detection of Methicillin Resistance in Isolated Bacteria

Methicillin resistance was confirmed phenotypically using the following methods:

Vitek2 system: This system was used to detect resistance to cefoxitin and oxacillin.

Oxacillin Salt Agar Screen Test: Mueller-Hinton agar (Lab M / UK) plates containing 4% NaCl and 6 µg/ml of oxacillin powder were prepared. Plates were inoculated with 1 µl of a 0.5 McFarland suspension of the isolate, streaking in one quadrant, and then incubated at 33 to 35° C for 24 hrs. Testing at temperatures above 35°C may not detect methicillin-resistant strains. The plates were carefully examined under transmitted light for the presence of multiple colonies or a light film of growth. Accordingly, more than one colony illustrated oxacillin resistance [16].

PBP2a Screening test: This assay was performed using the Penicillin-Binding Protein (PBP2a) Latex Agglutination Test kit (Oxoid, Hampshire, UK) according to the manufacturer’s instructions.

### 2.7 Extraction of Genomic DNA

Genomic DNA was extracted from clinical isolates using the EzWay™ bacterial genomic DNA kit (Komabiotech, South Korea). The quality and quantity of the extracted DNA were also confirmed using a NanoDrop (Thermo Fisher Scientific, USA) by measuring the optical density at 260 and 280 nm.

### 2.8 Identity Confirmation by Polymerase Chain Reaction (PCR)

To further confirm the identity of bacterial isolates, specific primers (Table **1**) were designed and used in PCR. Therefore, the 16S rRNA, nuc, and mecA genes were used to confirm the *Staphylococcus genus, S. aureus* species, and methicillin resistance, respectively [17]. Multiplex PCR was performed in a final volume of 25 μL, consisting of 12.5 μL of master mix, 1.5 μL of each primer (three pairs for all genes), and 3.5 μL of template DNA.

### 2.9 Pulsed Field Gel Electrophoresis

For bacterial genotyping, all samples were sent to the laboratory at the College of Medicine, Cukurova University, Adana, Turkey [20]. Pulsed-field gel electrophoresis (PFGE) was used to assess the genetic relationship between *Staphylococcus isolates* from diabetic foot infections and the nares of the patients. Banding patterns were compared according to standard criteria, with ≥80% similarity considered indicative of clonal relatedness.

### 2.10 Data Analysis

All experiments were performed in triplicate. The data were expressed as mean ± SD, and all statistical analyses were conducted using SPSS version 23.

## 3 RESULTS

### 3.1 Profile of Bacteria Isolated from Diabetic Foot Patients

Gram-positive bacteria, including 22 genera/species, were isolated from diabetic foot wounds (Table **2**). The most frequent isolated bacterium in this study was *S. aureus* (33.1%).

### 3.2 Profile of Bacteria Isolated from the Nares of Patients

Of the 84 nasal swabs collected, 51 (60.7%) showed positive culture growth on Mannitol Salt Agar (MSA) media, as mentioned in Table **3**. Among these, 36 isolates were mannitol non-fermenters and were excluded from further identification using the Vitek2 system. Of the 15 mannitol-fermenting isolates analyzed by Vitek2, only five were identified as *Staphylococcus aureus*. The remaining isolates were coagulase-negative *Staphylococci* (CoNS), which can also ferment mannitol on MSA, as reported previously [21].

### 3.3 Antibiotic Resistance Pattern

Our data (Table **4**) demonstrated that 55.1% of the Gram-positive isolates were resistant to methicillin. Resistance to erythromycin, tetracycline, and levofloxacin was 50.6%, 48.3%, and 41.6%, respectively. In contrast, the lowest resistance rates were observed for vancomycin (3.4%), mupirocin (4.5%), and linezolid (7.9%).

Among the 15 *Staphylococcus* isolates obtained from the nares of diabetic patients, resistance to 16 antibiotics was as follows: eleven isolates were resistant to methicillin (cefoxitin screen), 10 to tetracycline, 9 to erythromycin, and 7 each to levofloxacin and clindamycin (Table **5**).

### 3.4 Further Confirmation of Methicillin Resistance in *Staphylococci*

In addition to the Vitek2 system, two other phenotypic assays were employed to confirm methicillin resistance in *Staphylococcus* isolates. The Oxacillin Salt Agar Screen test detected resistance in 78.5% of the isolates, while the PBP2a assay detected resistance in 75.4%, with no significant difference observed between the two methods.

### 3.5 Molecular Identification of Isolated Bacteria

Two species-specific genes, 16S rRNA and nuc, were analyzed by PCR to confirm the identity of *Staphylococcus* bacteria and *S. aureus* species, respectively. As shown in Fig. (**1A**), all *Staphylococcus* species were positive for 16S rRNA, except for *S. lentus* from patient 46. Similarly, the PCR amplicon for the nuc gene was detected in all *S. aureus* isolates, as shown in Fig. (**1B**). These results were further confirmed by multiplex PCR (Fig. **1C**).

### 3.6 Molecular Confirmation of Methicillin Resistance by PCR

PCR was employed to detect the presence of the mecA gene in all isolates. Our results showed that 95.3% of *Staphylococcus* isolates were methicillin-resistant (Fig. **1C**). Among the 30 *S. aureus* isolates, only two were negative for the mecA gene. Similarly, of the 27 coagulase-negative *Staphylococci* (CoNS) isolates, 26 were methicillin-resistant.

### 3.7 Comparison between MSSA and MRSA in the *mecA* Gene Harboring

Among the 31 methicillin-susceptible *S. aureus* (MSSA) isolates, 9 (29.0%) harbored the mecA gene, whereas 20 (64.5%) of the 21 methicillin-resistant *S. aureus* (MRSA) isolates carried the mecA gene (Table **6**).

### 3.8 Comparison of the mecA Gene Carriage between Patients with and without Prior Antibiotic Use

The mecA gene was detected in a higher number of *Staphylococcus* isolates obtained from patients with a history of prior antibiotic use compared to those without such a history (Table **7**).

### 3.9 Similarity between *Staphylococci* Bacteria Isolated from the Nares and the Foot of the Same Diabetic Foot Patient

Using the VITEK 2 system, similarity between *Staphylococcus aureus* isolates from the nares and foot of the same patient was observed in both identity and antimicrobial susceptibility patterns. A similar observation was made for a coagulase-negative *Staphylococcus* (CoNS, S. lentus) isolated from both the nares and foot of the same patient (Table **8**).

Confirmation of similarity was performed for two *S. aureus* isolates and one CoNS (*S. lentus*) using pulsed-field gel electrophoresis (PFGE) (Fig. **2**). The results demonstrated 100% genetic similarity between isolates from the same patient, as illustrated by the dendrogram in Fig. (**3**).

Regarding methicillin susceptibility (Table **9**), the *S. aureus* isolates were phenotypically methicillin-sensitive but carried the mecA gene genotypically. In contrast, *S. lentus* was phenotypically methicillin-resistant and also harbored the mecA gene genotypically.

## 4 DISCUSSION

Many studies have investigated the bacteriology of diabetic foot infections, but results vary across reports. In this study, only a small proportion of patients with diabetic foot ulcers had negative culture results, while the majority of patients harbored bacteria in their wounds [22-24].

Resistance to several antibiotics was high, including erythromycin, tetracycline, levofloxacin, clindamycin, trimethoprim/sulfamethoxazole, rifampicin, and gentamicin [25]. Resistance was also observed for antibiotics typically considered to have low or no resistance, such as tobramycin, moxifloxacin, linezolid, teicoplanin, tigecycline, and mupirocin. This widespread resistance contributes to the emergence of multidrug-resistant (MDR) strains and limits treatment options [26]. Based on our findings, some antibiotics frequently used empirically in our region may no longer be fully effective, and clinicians should consider alternative or combination therapies.

The prevalence of mecA genes in *S. aureus* was alarmingly high in diabetic foot infections in this study [27]. Notably, there was discordance between phenotypic and genotypic results; some phenotypically MSSA strains were mecA-positive, while one MRSA strain was mecA-negative. This discrepancy may be explained by the involvement of genes other than mecA in resistance mechanisms, hyper-production of beta-lactamase enzymes [28], or limitations of the cefoxitin test in detecting low-level methicillin resistance [29].

Methicillin resistance in CoNS was also found to be alarmingly high, both phenotypically and genotypically. The mecA gene is located on a mobile genetic element, the *Staphylococcal* cassette chromosome mec (SCCmec), which is widely disseminated among *Staphylococcus* species [30]. This may be due to horizontal transfer of this element between staphylococcal species [31, 32].

Comparison of the prevalence of antibiotic-resistance genes with patients’ prior antibiotic use revealed that isolates from patients with a history of antibiotic exposure had a higher percentage of resistance genes than those from patients without such a history [33, 34]. This suggests that prolonged antibiotic exposure promotes the development of resistance. Notably, CoNS strains exhibited higher resistance to most antibiotics compared to *S. aureus*. This finding is consistent with Sangeda *et al.*, who suggested that the mec gene responsible for *S. aureus* resistance may have originated from other staphylococcal species [35].

In this study, the genetic relationship between two *S. aureus* strains and one CoNS strain isolated from diabetic foot ulcers and the nares of the same patients was confirmed by PFGE. Our findings suggest that the source of foot ulcer contamination may sometimes be the patient’s nasal flora. Eradication of staphylococci from foot ulcers may be difficult if the nasal reservoir remains colonized. Nasal carriage of *S. aureus* has been identified as a significant risk factor for nosocomial and surgical site infections [34, 36-40]. However, the link between nasal carriage of *S. aureus* or CoNS and diabetic foot infections remains poorly understood [4, 6, 7, 9, 41]. Screening for and eliminating nasal carriage is therefore an important step to reduce the risk of *staphylococcal* infections, particularly methicillin-resistant strains, in foot ulcers [42].

In the present study, only selected genetic markers were used to assess relationships among isolates. Whole-genome sequencing or more advanced molecular typing could not be performed, which might have provided a more comprehensive understanding of potential genetic correlations among isolates. Additionally, the lack of follow-up limited the ability to determine whether nasal carriage contributed to recurrent or persistent infections in the same patients.

## CONCLUSION

In conclusion, among 40 patients, 22 (55%) had *staphylococcal* isolates, of which 31.8% showed genetic similarity between nasal and wound samples. Confirmation of similarity between bacteria isolated from the wounds and nares of the same patient by PFGE supports the hypothesis of auto-infection *via* mechanical transfer of bacteria to the wounds. Routine nasal screening of diabetic patients who are nasal carriers, strict infection control measures, and antibiotic stewardship programs are recommended to reduce transmission and prevent the development of resistant strains.

## Figures and Tables

**Fig. (2):**
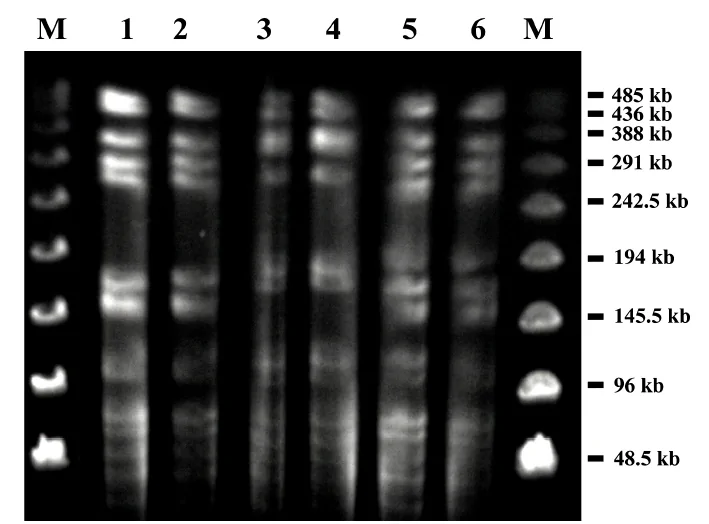
Pulsed-field gel electrophoresis (PFGE) of genomic DNA from six bacterial isolates from the nares and foot of three patients. M: Lambda PFG ladder; lanes 1-6: bacterial isolates.

**Fig. (3):**
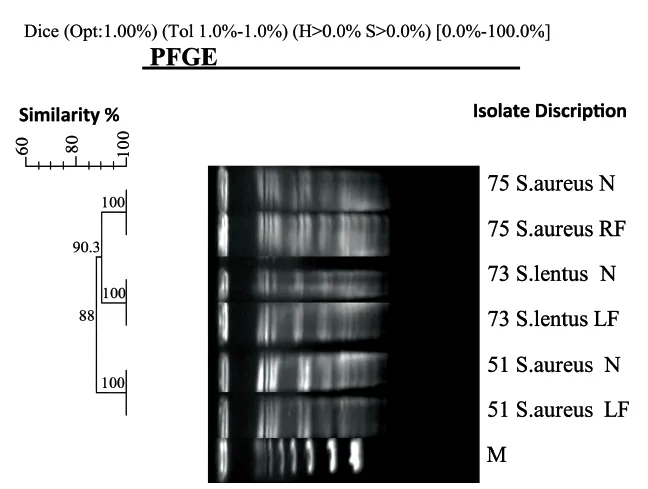
Dendrogram showing genetic similarity between isolates from the nares and foot of three patients based on PFGE of SmaI-digested genomic DNA.

**Fig. (1):**
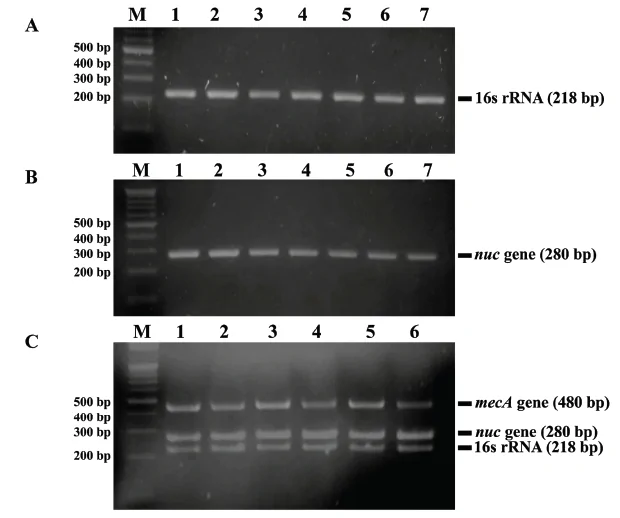
(**A**) PCR amplification of the 16S rRNA gene in *Staphylococcus* species. Lanes 1-7 show PCR amplicons from all samples. (**B**) PCR amplicons of the nuc gene in *S. aureus*. (**C**) Multiplex PCR for all genes studied in this project. M: Marker (100 bp).

**Table 1:** Primers used to detect bacterial identity and methicillin-resistance genes.

**Primer Name**	**Primer Sequence**	**Amplicon Length (bp)**	**References**
*16S rRNA-F* *16S rRNA-R*	5´-GTAGGTGGGAAGCGTTATCC-3´ 5´-CGCACATCAGCGTCAG-3´	218	[18]
*nuc gene-F* *nuc gene-R*	5´-GCGATTGATGGTGATACGGTT-3´ 5´-AGCCAAGCCTTGACGAACTAAAGC-3´	280	[18]
*mecA*-F *mecA*-R	5´-CTCAGGTACTGCTATCCACC-3´ 5´-CACTTGGTATATCTTCACC-3´	448	[19]

**Table 2:** Profile of bacteria isolated from foot wounds of diabetic patients.

**Bacteria Name**	**Value No. (%)**
*Staphylococcus aureus*	33 (37.1)
*Staphylococcus haemolyticus*	12 (13.5)
*Kocuria kristinae*	10 (11.2)
*Staphylococcus lentus*	6 (6.7)
*Enterococcus faecalis*	6 (6.7)
*Staphylococcus epidermidis*	4 (4.5)
*Staphylococcus xylosus*	2 (2.2)
*Staphylococcus intermedius*	2 (2.2)
*Staphylococcus chromogenes*	1 (1.1)
*Staphylococcus gallinarum*	1 (1.1)
*Staphylococcus hominis ssp hominis*	1 (1.1)
*Staphylococcus pasteuri*	1 (1.1)
*Staphylococcus pseudointermedius*	1 (1.1)
*Staphylococcus saprophyticus*	1 (1.1)
*Staphylococcus warneri*	1 (1.1)
*Streptococcus pneumoniae*	1 (1.1)
*Streptococcus agalactiae*	1 (1.1)
*Kocuria rosea*	1 (1.1)
*Kocuria varians*	1 (1.1)
*Kytococcus sedentarius*	1 (1.1)
*Dermacoccus nishinomiyaensis*	1 (1.1)
*Rothia dentocariosa*	1 (1.1)

**Table 3:** Profile of bacterial isolated from the nares of diabetic food patients.

**Bacteria**	**No. (%)**
**Mannitol non-fermenter**	36 (70.6)
**Mannitol fermenter**	15 (29.42%)
*Staphylococcus aureus*	5 (9.8)
*Staphylococcus haemolyticus*	4 (7.8)
*Staphylococcus lentus*	2 (3.9)
*Staphylococcus lugdunensis*	2 (3.9)
*Staphylococcus saprophyticus*	1 (2.0)
*Staphylococcus pasteuri*	1 (2.0)

**Table 4:** Antibiotic resistance patterns of all Gram-positive bacteria to 16 antibiotics.

**Antibiotic**	**No. of Resistant Isolates (%)**
Cefoxitin Screen	49(55.1)
Gentamicin	22(24.7)
Tobramycin	20(22.5)
Levofloxacin	37(41.6)
Moxifloxacin	14(15.7)
Inducible Clindamycin Resistance*	10(11.2)
Erythromycin	45(50.6)
Clindamycin	35(39.3)
Linezolid	7(7.9)
Teicoplanin	12(13.5)
Vancomycin	3(3.4)
Tetracycline	43(48.3)
Tigecycline	6(6.7) NS*
Mupirocin	4(4.5) LR*
Rifampicin	24(27.0)
Trimethoprim/Sulfamethoxazole	32(36.0)

**Note:** *NS* = Non-susceptible, *LR* = Low resistance.

**Table 5:** Antibiotic resistance patterns of *Staphylococcus* isolates from the nares of diabetic foot patients to 16 antibiotics.

**Antimicrobial Agent**	**No. of Resistant Isolates (%)**
Cefoxitin Screen	11(73.3)
Gentamicin	4(26.7)
Tobramycin	3(20.0)
Levofloxacin	7(46.7)
Moxifloxacin	3(20.0)
Inducible Clindamycin Resistance	3(20.0)
Erythromycin	9(60.0)
Clindamycin	7(46.7)
Linezolid	0(00.0)
Teicoplanin	2(13.3)
Vancomycin	0(00.0)
Tetracycline	10(66.7)
Tigecycline	2(13.3)NS*
Mupirocin	0(00.0)
Rifampicin	3(20.0)
Trimethoprim/Sulfamethoxazole	6(40.0)

**Note:** *NS* = Non-susceptible.

**Table 6:** Comparison of mecA gene carriage between MSSA and MRSA isolates.

***S. aureus***	***mecA* Positive**	***mecA* Negative**	**Total**
MSSA	9(29.0%)	1(3.2%)	10(32.3%)
MRSA	20(64.5%)	1(3.2%)	21(67.7%)
**Total**	29((93.5%)	2(6.4)	31(100%)

**Table 7:** Presence of mecA genes in *Staphylococcus* isolates from patients with and without prior antibiotic use.

**Gene**	**No Previous Antibiotic Use**		**Previous Antibiotic Use**		**Total**
	**Gene Detected**	**Gene Not Detected**	**Gene Detected**	**Gene Not Detected**	
*mecA*	14(23.0%)	0(0.0%)	44(72.1%)	3(4.9%)	61(10)

**Table 8:** Similarity between *Staphylococcus* isolates from the nares and foot of the same patient.

**Pt. Code**	**Bacteria**	**Location of Isolation**
51	*Staphylococcus aureus*	Left foot + Nose
75	*Staphylococcus aureus*	Right foot +Nose
73	*Staphylococcus lentus*	Left foot +Nose

**Table 9:** Methicillin susceptibility patterns of bacteria isolated from the nares and foot of the same patient.

**No**	**Patient Code**	**Bacteria**	**Methicillin Susceptibility Test Results**			***mecA* Gene**
			**VITEK 2 System**	**Oxacillin Salt-Agar Screen Test**	**PBP2a Test**	
1	51N	*S. aureus*	Negative	Negative	Negative	Positive
2	51LF	*S. aureus*	Negative	Negative	Negative	Positive
3	75N	*S. aureus*	Negative	Negative	Negative	Positive
4	75RF	*S. aureus*	Negative	Positive	Positive	Negative
5	73N	*S. lentus*	Positive	Positive	Positive	Positive
6	73LF	*S. lentus*	Positive	Positive	Positive	Positive

## Data Availability

The data used to support the findings of this study are available from the corresponding author [S.H.] upon request.

## LIST OF ABBREVIATIONS

MRSA: Methicillin-resistant *Staphylococcus aureus*
CoNS: Coagulasenegative Staphylococci
PFGE: Pulsed-field Gel Electrophoresis

## STANDARDS OF REPORTING

STROBE guidelines were followed.
