# Supplementary material for: Characteristic Correlation between Staphylococcal Isolates from Wounds and Nares of Diabetic Foot Patients Living in Erbil City, Iraq
Source: Curr Diabetes Rev. 2026 Mar 26;22(9):E15733998413217. doi: 10.2174/0115733998413217251206210451 (PMC13725160; doi:10.2174/0115733998413217251206210451)
Supplement: Supplementary file 1 [file CDR-22-9-E15733998413217_SD1.pdf]

Supplementary Material

Characteristic Correlation between Staphylococcal Isolates from Wounds and Nares of Diabetic Foot Patients Living in Erbil City, Iraq

Samira Fattah Hamid[1,*], Fatih Koksal[2], Tulin Guven Gokmen[3] and Tulay Knademir[2]

[1]Department of Medical Microbiology, College of Health Sciences, Hawler Medical University, Erbil, Iraq; [2]Department of Clinical Microbiology, Faculty of Medicine, Cukurova University, Adana, Turkey; [3]Department of Microbiology, Ceyhan Veterinary Faculty, Cukurova University, Adana, Turkey

Table S1. Summary of bacteria isolated from nasal and foot samples of 84 patients.

Patients	Nasal Swab	Foot Swab	Gram Positive Isolated from Foot	Nasal Positive Culture	Genetic Correlation
84	84	84	89 bacteria (22 genera/species)	51 bacteria (36 mannitol fermentor + 15 non mannitol fermentor)	3 match 100%
